# A randomized controlled trial of suicide prevention training for primary care providers: a study protocol

**DOI:** 10.1186/s12909-019-1482-5

**Published:** 2019-02-14

**Authors:** Wendi F. Cross, Jennifer C. West, Anthony R. Pisani, Hugh F. Crean, Jessica L. Nielsen, Amanda H. Kay, Eric D. Caine

**Affiliations:** 1 Department of Psychiatry, University of Rochester Medical Center, Rochester, NY 14642 USA; 2 Injury Control Research Center for Suicide Prevention, Rochester, NY 14642 USA; 3 School of Nursing, University of Rochester, Rochester, NY 14642 USA

**Keywords:** Suicide prevention, Adolescents, Medical residency education, Primary care, Telehealth, Simulation

## Abstract

**Background:**

Suicide is a national public health crisis and a critical patient safety issue. It is the 10th leading cause of death overall and the second leading cause of death among adolescents and young adults (15–34 years old). Research shows 80% of youth who died by suicide saw their primary care provider within the year of their death. It is imperative that primary care providers develop the knowledge and skills to talk with patients about distress and suicidal thoughts, and to assess and respond in the context of the ongoing patient - primary care provider relationship.

**Methods:**

This study examines the effectiveness of simulation on suicide prevention training for providers-in-training by comparing two conditions: 1) a control group that receives online teaching on suicide prevention in primary care via brief online videos and 2) an experimental group that includes the same online teaching videos plus two standardized patient (SP) interactions (face-to-face and telehealth, presentation randomized). All SP interactions are video-recorded. The primary analysis is a comparison of the two groups’ suicide prevention skills using an SP “test case” at 6-month follow-up.

**Discussion:**

The primary research question examines the impact of practice (through SP simulation) over and above online teaching alone on suicide prevention skills demonstrated at follow-up. We will assess moderators of outcomes, differences among SP simulations (i.e., face-to-face vs. telehealth modalities), and whether the experimental group’s suicide prevention skills improve over the three SP experiences.

**Trial registration:**

The study was registered on Clinical Trials Registry (clinicaltrials.gov) on December 14, 2016. The Trial Registration Number is NCT02996344.

## Background

Suicide is a national public health crisis and a critical patient safety issue. Suicide is the 10th leading cause of death overall and the second leading cause of death in adolescents and young adults (15–34 years old) [1]. For every death by suicide there are approximately 25 suicide attempts that do not result in death [2]. Individuals who survive suicide attempts ultimately experience much higher rates of mental and physical health issues compared to non-attempters [3]. Despite a variety of national prevention efforts including the Surgeon General’s National Strategy for Suicide Prevention, suicide rates, including among youth and young adults, have climbed over the past two decades [4].

A recent U.S. study of suicide reported that 45% of individuals who died by suicide had contact with primary care services within one month before their death [5]. Another study found that 80% of youth who died by suicide saw their primary care provider within a year of their death [6]. Suicidal patients are more likely to see a primary care provider than a mental health professional. In fact, only 20% of individuals who died by suicide saw a mental health provider within a month of their death, compared to 45% of suicidal individuals who saw a primary care provider within a month of their death [5, 7].

Training health care providers is likely to reduce suicide attempts and deaths [8], and suicide is deemed a “Never Event” by the National Quality Forum. Yet training in suicide prevention is not required in most medical and post-graduate education programs [9] and only a few states require it for licensure. Where training does exist, its effectiveness has not been studied and there is no consensus on the best methods for improving the care of suicidal patients.

One promising methodology for training providers in suicide assessment and safety planning skills is simulation. There is a long history of simulation-based learning methods in medical [10] and nursing school [11] training programs. Since 1963, standardized patients (SPs; specially-trained actors who present patient scenarios, symptoms or signs and feedback) have been used as an active learning education tool for teaching and learning. Systematic reviews have shown evidence for improvements in learners’ knowledge and confidence with high fidelity simulation methods [12, 13] including resident screening for adolescent depression [14]. Feedback from simulated patient interactions has been used successfully in psychiatry resident education to teach suicide assessment skills [15]; however, these methods have not been applied to other health professionals such as primary care providers, and the effect of standardized patient practice has not been directly measured in a controlled study.

Another challenge for medical education programs is to prepare trainees for technological advances in health care, including rapidly increasing use of telemedicine contact with patients via telephones, smartphones, and mobile wireless devices, with or without a video connection [16, 17]. Patients living in rural communities and those with mental health needs are particularly likely users for telehealth care [18, 19]. There are currently barriers to routine telehealth practice (e.g., reimbursement, access); however, given the trends in healthcare, studies on ‘remote’ provider-patient interactions are critical. To our knowledge, there are no studies of telehealth-based suicide risk assessment and management.

The current study responds to the need for effective suicide prevention training for primary care providers [11]. The primary research question asks whether experiential and reflective practice, via standardized patient simulation and feedback, improve suicide prevention skills over and above expert teaching alone. We test simulation as a component of the training because, while resource-intensive, it involves techniques that are core tenets of adult learning and is highly likely to be effective [20]. We incorporate specific feedback into the training as it has been shown to be a key component of learning new skills in the medical field [21]. Assurance of providers’ ability to identify and effectively respond to those who are suicidal is an ideal use of simulation-based training and assessment methods [22]. We include telehealth as a modality in the experimental condition with the knowledge that it will soon be a normative practice for primary care providers and to contribute to our understanding of interventions with suicidal patients.

This randomized control trial (RCT) examines the effectiveness of simulation and feedback, using standardized patient (SP) interactions, on the suicide risk assessment and management skills of primary care providers-in-training. There are two groups: 1) a control group that receives online teaching delivered via brief videos and, 2) an experimental group that receives the same brief videos followed by two SP practice experiences with feedback – one face-to-face and one telehealth. Both groups’ skills are tested via an in-person SP “test case” at 6-month follow up. All SP interactions are followed by immediate feedback given by the SP that is specifically linked to training concepts.

Hypotheses are as follows: 1) All participants will gain suicide prevention knowledge from baseline after viewing online teaching and will maintain improvement at 6-month follow-up; 2) Participants in the experimental group will report greater satisfaction with the training, greater self-efficacy in identifying and responding to patients with suicidal thoughts and plans, and greater intention to use, as well as reported use of suicide prevention skills at 6-month follow-up; and 3) Participants in the experimental group will also be more skillful in responding to suicidal patients (demonstrated during the SP “test case” interview as measured by objective, observed ratings) compared to the control group at 6-month follow-up. Additionally, we will examine moderators of outcomes, differences between the two SP simulation modalities (i.e., face-to- face vs. telehealth modalities), and if there is improvement in skills observed over multiple SP interactions for the experimental group.

## Methods

### Aim

This study aims to compare online teaching alone to online teaching plus practice through simulation on the suicide risk assessment and safety planning skills of primary care providers-in-training.

### Design

The study is a randomized controlled trial with an experimental group and a control group allocated with a 1:1 randomization ratio. All trainees are randomized to control and experimental learning groups using Wei’s urn randomization [23] which provides overall balance at the end of accrual but also gives good, often near-perfect, balance within many strata [24, 25]. Randomization strata include medical learner groups (i.e., medical residents, pediatric residents, and nurse practitioners) and gender. After all baseline measures are completed, participants are randomized to experimental condition by the project statistician, who remains blinded to study condition.

Participants in both groups complete a 48-min, 6-module, online video-based training on suicide prevention (Commitment to Living for Primary Care; CTL-PC). Upon completion of the online teaching program, participants in the experimental group engage in two standardized patient practice interactions over the course of 4 to 6 months. One practice interaction takes place in-person and the other uses a telehealth model. The order is randomized. Then, approximately 6 to 8 months (depending on schedules, see below) after video-based learning, both control and experimental groups engage in an in-person “test case” standardized patient interaction.

### Setting

The suicide prevention training occurs in the context of pediatric and family medicine residency and nurse practitioner training programs at the University of Rochester Medical Center. SP sessions are conducted at the convenience of the participants at several medical training sites including the University of Rochester School of Nursing, Strong Memorial Hospital, the Department of Family Medicine, and the Rochester General Pediatric Associates. Each SP interaction takes place in a private room and the entire session is a maximum of one hour in duration. In the case of telehealth interactions, standardized patients are in a different location from participants.

### Participants

Participants are second year trainees from two education programs at the University of Rochester Medical Center: the nurse practitioner training program (NP) and medical residency programs, which included second year residents from pediatrics, the combined internal medicine-pediatrics program, and family medicine. These learner groups were chosen because most are preparing for careers in primary care. The training directors of the programs agreed to incorporate suicide prevention training into their curriculum for the duration of the grant and to support randomization of learners. As a result, all trainees receive the training according to their randomized condition. Trainees engage in a consent process (conducted by study personnel) for analysis of their data; those who do not consent for the study receive the training but their data are not analyzed. All trainee participants are fully informed that the SP interactions are part of their suicide prevention training. We collect non-identifiable demographic data at baseline for all trainees in order to compare those who enrolled in the study and those who did not. We plan to enroll 108 participants generally divided between residents and NPs.

### Online teaching

“Commitment to Living for Primary Care” (CTL-PC) was adapted for primary care from an evidence-based suicide prevention training program for mental health professionals that has been widely disseminated [26, 27]. The online teaching was presented by one of us (AP) who has had more than a decade’s experience training clinical staff in the evaluation of suicidal persons. CTL-PC consists of six brief video modules (48 min in total) that focus on practical aspects of person-centered care for patients with suicidal thoughts and plans. The modules are as follows: introduction to suicide prevention in primary care; person-centered approach to asking about suicide in primary care; gathering data to inform risk assessment; synthesizing data into a formulation of risk; responding to acute and ongoing risk in primary care; and special considerations (adolescents, substance abuse, intimate partner violence, and LGBT).

### Procedures

Following enrollment, participants receive an email with instruction to complete online baseline assessments and view the teaching videos within 14 days. All surveys are completed online using the same secure website that hosts the CTL-PC videos. After they have viewed the videos, participants in the experimental condition engage in two practice SP interactions over a period of about four months, with at least one month between interactions. Participants in the control condition do not have practice interactions. At approximately six months following completion of online teaching, both experimental and control participants complete the same face-to-face SP test case. There are two SP character scenarios for the experimental practice sessions and one test case SP scenario.

As part of their practice experience, experimental participants engage in telehealth and face-to-face SP interactions; the order of presentation is randomized. The SP practice character scenario is also randomized (male/female) for the experimental group. All interactions are video-taped for observational coding of suicide prevention skills. In the telehealth modality, the participant and standardized patient are in different locations and interact using a secure webcam service. The face-to-face modality occurs in an office setting. The procedures for face-to-face and telehealth interactions are the same and no more than one hour in duration, including: orientation to the procedures and the SP scenario backstory, up to 30 min for the SP interaction, 8 min for SP feedback for the participant, and 5 to 10 min for debrief procedures. Upon arrival to the interaction, participants are introduced to the procedures and are given a brief written backstory about the SP scenario. Backstories provide context for the provider and include details about the patient’s name, age, the nature of the patient-provider relationship, some medical and social history and the reason for this visit (“trouble sleeping”). Telehealth participants also receive instructions about remote access. Participants are instructed to assess the patient’s risk for suicidality and provide appropriate interventions within a 30-min timeframe (a timer signals the appointment end). After the interaction, participants exit the room for 10 min during which time the SP prepares immediate feedback (positive and constructive) for the patient. SPs are highly trained to prepare and deliver tailored feedback using a 15-item “feedback matrix” template of criteria for identifying appropriate feedback items. Feedback includes specific competency items with explanations and rationale for the importance of the skill. The participant joins the SP for 8 min to receive feedback, discuss the interaction and ask questions. Afterwards, the participant returns to the facilitator for 5 to 10 min of debrief procedures. During this time, participants are provided the opportunity to speak with a psychologist for further debriefing if needed.

All interactions are recorded, securely stored and subsequently coded for observed suicide prevention skills. A portion (at least 20%) of recorded interactions are double coded for inter-rater reliability. Consensus meetings are conducted and discrepancies are resolved through discussion. Additionally, the videos are coded for SPs fidelity to the scenario and feedback procedures. To avoid drift in standardized procedures, SPs receive written feedback on fidelity to the character and feedback delivery.

After the SP test case at approximately 6-month follow-up, all participants complete a final survey including: knowledge and self-efficacy measures, retrospective self-report of transfer of training, intentions to use the suicide prevention training in future practice, self-reported use of suicide prevention skills since the online teaching program, and satisfaction with various aspects of the training.

### Measures

#### Self-report measures

##### Demographics

All participants complete a survey of individual characteristics and demographics, including training program (residency, NP), gender, age, previous suicide prevention training, previous experience with SP procedures, and prior experience (professionally and personally) with suicide. These data will be examined to determine any differences between those who consent for study participation and those who do not, and be used for analytic purposes.

##### Knowledge of suicide and suicide prevention

Participants complete a 17-item multiple choice knowledge assessment before and after viewing online teaching videos and again at 6-month follow-up. The measure assesses knowledge of suicide risk assessment and management as covered in the online teaching program (e.g., “Which of the following best describes risk for suicide?”). A measure of knowledge has been used in published assessments of the CTL training curriculum [27] with good internal consistency (Cronbach’s α = .81–.87) and sensitivity to change (Cohen’s d = .90–1.35). For the purposes of the current study modifications will be made to the measure.

##### Suicide prevention self-efficacy

Participants complete a 20-item measure of self-efficacy at pre- and post-online teaching and again at 6-month follow-up. The measure uses an 8-point Likert scale (ranging from “strongly disagree” to “strongly agree”) to assess trainees’ self-efficacy regarding suicide risk assessment, treatment, and documentation (e.g., “I feel confident that I can ask directly about suicide,” “I feel confident in my knowledge about the key elements of a safety plan.”) The original CTL self-efficacy measure [27] has acceptable internal consistency (Cronbach’s α = .95–.96) and sufficient sensitivity to change over time (Cohen’s d = 1.05–1.15); modifications will be made for the current study.

##### Transfer of training

We also assess participants’ perception that they will transfer what they learned in the online program into clinical practice using an 8-item measure modified from three subscales (transfer design, perceived content validity, opportunity to use) of the Learning Transfer System Inventory [28]. The LTSI subscales have been shown to differentiate trainees who will ultimately use newly trained skills in practice [29]. At post-online teaching and at the 6-month follow up, participants rate items (e.g., “I will be able to use this training on my job,” “It is clear to me that the developers of the training understand how I will use what I learn”) on a 5-point Likert scale (from “strongly disagree” to “strongly agree”). The total of the 8 items will be used as a composite transfer of training score. In addition to being a valid instrument, the LTSI has good internal reliability (Cronbach’s α = 85) [27, 29].

##### Use of suicide prevention skills in practice

At the 6-month follow up, participants complete a 4-item measure rated on a 5-point Likert scale (“not at all” to “frequently”) to assess how often they used the suicide prevention skills taught in the training (e.g., “Since the CTL- PC videos/online teaching program, I have used the suicide prevention skills.”).

##### Satisfaction with suicide prevention training

At 6-month follow up, participants complete a 12-item measure of satisfaction with the training using a 5-point Likert scale (“not at all satisfied” to “very satisfied”). Participants are asked about their satisfaction with aspects of the training videos, (e.g., quality, duration, accessibility, and amount of information), as well as with their SP experience(s) (e.g., realism, quality of feedback).

#### Observational ratings

##### Observational coding

Video recordings of SP practice interactions and SP test case interactions for all participants are coded by a research team using an objective observational coding measure with five domains of skills related to the training: non-anxious and empathic presence, assessment of risk factors, assessment of protective factors, asking about suicidal ideation and behavior, and safety planning. Initial development work established acceptable inter-rater reliability using Gwet’s AC1 statistic, which is considered preferable to Cohen’s Kappa in cases when raters produce ratings that are not necessarily statistically independent [30]. Participants are also rated (“needs work,” “acceptable work,” and “good work”) on a general quality item (e.g., degree of collaboration, tone).

##### Feedback matrix

Immediately following each interaction, SPs prepare feedback for the participant on 15 items and document the ratings on a Scantron-style form. SPs verbally provide participants with six feedback items – three positive and three constructive. Several items are prioritized as feedback items to provide to the participant. In the case of experimental participants’ second and final sessions, SPs have information on the feedback items that have been previously provided to the participant as guidance for novel feedback.

##### Standardized patient fidelity

Coders assess SP fidelity to the character scenario and procedures using a 9-item actor fidelity measure coded as “present,” “absent,” or “not-applicable.” For example, the SP is rated on affect (displays affect consistent with character) and sequence and timing of disclosure (“sometimes I wonder if I should end it all” within 7 min if not already asked directly).

#### Materials

##### Equipment

In-person sessions are recorded with a webcam and microphone using computer software called Debut Video Capture. Telehealth sessions are also recorded with the same equipment using a secure video conferencing and recording software (Zoom). Recordings are uploaded to a secure website for storage and access for coding.

##### Standardized patient scenarios

Three scenarios were developed for the study with input from subject matter experts. Each scenario features an older adolescent/young adult primary care patient experiencing suicidal thoughts in the context of stressful life events. The three scenarios were matched for upstream factors (e.g., life stressors, social support) and difficulty. A male and a female scenario are modified slightly for telehealth interactions (experimental condition). The scenario used for the SP test case that all participants receive is an in-person female scenario. Choice of female gender for the test case reflects the greater proportion of suicide attempts in females.

### Statistical analysis

#### Data processing

Data collection and storage is managed by REDCap, a secure software system supported by the institution. (Data management procedures are fully described in the study manual.) A baseline check of randomness will be conducted for each demographic and outcome variable collected. Because we are interested in establishing equivalence (as opposed to detecting differences), a more conservative alpha level will be applied (α = .20). Categorical variables will be analyzed using chi-square tests; continuous variable differences will be assessed using a general linear model approach (ANOVA). Any baseline difference will be controlled for statistically in all subsequent analyses. For analyses related to hypotheses, α = .05 and covariates will include stratification variables of gender and residency/NP training program, race/ethnicity, age, and the specific outcome baseline score as well as any other variables found to differ at baseline. Effect sizes (Hedge’s *g*) will also be examined for each of the study hypotheses.

#### Hypotheses and exploratory analyses

##### Hypothesis 1

All participants will demonstrate improved suicide prevention knowledge from baseline after viewing the online teaching videos. We anticipate that this improvement will be maintained at the 6-month SP assessment. We do not anticipate a difference between the two conditions on knowledge measures. Repeated measures analysis of variance will be used to examine change in knowledge scores. Time will be examined as a within-subject factor (pre, post-videos, and follow up) with group entered as a between-subjects factor. Specifically, we hypothesize an overall time effect but no group or group by time interaction effects. Tryon’s inferential confidence intervals (ICI) will be used to assess for equivalency across treatment groups immediately post videos and immediately after the SP test case [31, 32]. In general, these inferential confidence intervals are adjusted so that “statistical difference” equates to two inferential confidence intervals that do not overlap (i.e., abutting at the upper or lower bounds). Establishing traditional 95% confidence intervals about each of two means, and concluding that the means differ *p* < .05 if the two intervals do not overlap, constitutes a greater burden of proof of statistical difference than would a corresponding t-test. To establish equivalence, however, a “Minimum Important Difference” (MID) must be established a-priori. By establishing MID’s, a context for determining statistical difference, equivalence, and indeterminacy is provided. Statistical differences exist when ICI’s do not overlap; statistical equivalence exists when the maximum probable mean difference estimate provided by the ICI (maximum upper bound of mean 1 and 2 minus the minimum lower bound of mean 1 and 2) is less than the MID and statistical difference does not exist. Indeterminacy exists when the means are neither statistically difference nor significantly equivalent. Following Treadwell and colleagues [33] a standardized small effect of .20 [34] will define the criterion for establishing a MID. As a form of sensitivity, a MID of .10 will also be examined.

##### Hypothesis 2

Participants in the experiment condition (simulation and feedback) will report greater self-efficacy in identifying and responding to patients at risk for suicide and greater intention to use the skills at 6 months compared to the control condition (those who only complete online teaching). We will use ANCOVA to assess SP training effects on satisfaction with the program, self-efficacy in identifying and managing patients who are at risk for suicide, and greater intention to use the skills at follow up. In all instances, the stratification variables of gender and training program group, as well as race/ethnicity/age and baseline status of the dependent variable (i.e., self-efficacy), will be included as covariates to aid in statistical power [35, 36]. If ceiling effects become an issue in any of these outcomes, tobit regression [37] will be used with the same covariates to assess for condition differences.

##### Exploratory analyses for Hypothesis 2

Moderation effects will be explored. The same ANCOVAs/tobit regressions described above will be conducted for each analysis but will also include interaction effects. Specifically, the cross-product of experimental condition by training group, baseline previous experience with suicidal patients, previous suicide prevention training, and baseline knowledge will be separately entered into each of the analyses. Procedures outlined by Aiken & West [38] will be used to minimize problems associated with multicollinearity. Multiplicative terms will be computed, first by centering each predictor variable and then forming the product term using the centered predictors of interest. Effect sizes, following Lipsey & Wilson [39], will also be examined as power to detect significant interactions will be limited [40]. The goals of these exploratory moderator questions are to better understand for whom the simulation training might be more effective (e.g., Do simulation training effects differ based on previous trainings/experiences with suicidal patients? Do simulation effects differ by training program group?).

##### Hypothesis 3

The primary hypothesis of the study is that participants in the experimental condition will demonstrate significantly better skills compared to the control group, as measured by reliable, observed ratings of behaviors in the SP assessment at 6 months. We will use ANCOVA to assess SP training effects at follow up on all participants’ skills. Skills will be measured by reliable observational ratings of several suicide specific and general interviewing skills. In all instances, gender, training program group, race/ethnicity, and age will be included as covariates to aid in statistical power [35, 36].

##### Exploratory analysis for hypothesis 3

We will examine statistical equivalence between the two SP modalities (face-to-face; telehealth). Similar to Hypothesis 1 (above), Tryon’s inferential confidence intervals will be used to assess for equivalency among face-to-face and telehealth modalities [31, 32]. Following Treadwell and colleagues [33], a standardized small effect of .20 [34] will define the criterion for establishing a MID. As a form of sensitivity, a MID of .10 will also be examined. As a second form of sensitivity, multilevel modeling will be used to decompose the variance associated with these observed training outcomes into variance attributed to the person as well as variance attributed to time (first SP interaction, second SP interaction) and variance attributed to modality. Since the order is randomized, we might expect more variance attributed to time (improving over time). Within these models, the learner (person) defines level 2 with the observed ratings being the dependent level one variables. Both time and modality will be entered as time varying covariates.

##### Exploratory analysis

We will analyze the observed skills for the experimental condition over the two practice SP interactions and examine if there is decay, maintenance or further growth in the test case scenario. Growth curve analyses will be used to test for linear change in observed suicide prevention skills across the three data collection points (two observations during a 4-month period and at the 6-month “test case” observation of all study participants at follow up). The HLM 7.0 statistical software package will be used for conducting growth curve and multilevel analyses [41]. Although attempts will be made to equate the time distance between observations for SP trainees, we do expect variability in the number of days between observations to exist across persons. Thus, time will be modeled to reflect change from baseline status (i.e., time 1 = 0; intercept corresponds to baseline status) and time will be a count of days from baseline to subsequent observations. Particular interest lies in the overall slope estimates (i.e., do skills change over time?) and predictors of change (slope). For example, the following questions will be addressed: “Does prior experience with suicidal individuals predict changes in skills over time?” Additionally, effect sizes (ES), based on the work of Feingold [42] will be examined. Estimates of the means at each time point are derived from the model and used for effect size calculations (mean difference divided by pooled standard deviation).

##### Power analyses

Optimal Design software [43] was used to assess statistical power. As previously stated, covariates will be included in all models to help improve power to detect a treatment effect [35, 36]. Power will be computed on a modest effect size estimate of .30. With a minimum final sample size of 108 participants and explaining 40% of the variance in the outcome, we have .51 power to detect an effect size of .30. However, power increases to .80 if 50% of the outcome variance is explained by covariates. If 60% of the outcome variance is explained via covariates, we have greater than .80 power to detect an effect size of .20. We believe that an effect size of .30 is attainable and we also expect that baseline values of the dependent variable of interest as well as other baseline variables are likely to account for 50% of the variance in the dependent variable. While we recognize that power will be more limited for the exploratory questions addressed (and will supplement all analyses with the use of effect sizes), we do feel that the knowledge gleaned will provide useful information with regard to dissemination of the simulation training — addressing whether implementation via telehealth is statistically equivalent to in-person implementation as well as providing preliminary knowledge about the amount and predictors of change over time.

##### Missing data

While we will make every effort to collect all data at each data collection point, missing data is likely. For analyses examining mean differences, pairwise deletion of missing data will be used, thus retaining all available information. As a form of sensitivity analyses, however, multiple imputation of missing data will be conducted. In short, multiple imputation uses a regression based approach to impute values for data that are missing. So that variance is not artificially constrained, multiple imputation incorporates random error into the imputation process [44, 45]. To improve estimates, the procedures put forth by Allison [46] regarding intervention studies will be followed. Here, each intervention condition is imputed separately and then the resulting datasets are merged. Following Rubin, [47] 10 imputations will be performed and each of the 10 resulting datasets will be analyzed as above. The results obtained from the 10 datasets will be combined following Rubin [47]. Multiple imputation has consistently demonstrated less biased estimates than most other traditional approaches to the handling of missing data (i.e., listwise, pairwise, mean imputation, single regression imputation) [44, 45, 48].

##### Type 1 error protection

To help control for Type 1 error, the Benjamin Hochberg (BH) method will be used to adjust for the multiple comparisons proposed in the current study [49]. The BH method adjusts for multiple comparisons by controlling false discovery rate instead of family-wise error rate. It is less conservative than the more traditional Bonferonni methods, yet still provides adequate protection against Type 1 error. Since its inception, there has been growing evidence suggesting that the BH method is the optimal solution to the multiple comparison problem in most practical situations.

## Discussion

This RCT examines the benefit of practice via realistic standardized patient (SP) simulation interactions with feedback as part of training in suicide prevention skills. Objective ratings of suicide risk assessment and safety planning for primary care providers-in-training will contribute to an evidence base for the effectiveness of simulation practice and feedback over and above video-based expert teaching. Given the data that show primary care is a critical site for suicide prevention, well trained and competent primary care providers are essential to reducing the prevalence of primary care patients who die by suicide.

Already, the logistics for scheduling the resident learner group has been an unanticipated, practical dilemma. It is important to note that the residency and NP training program directors agreed to embed the suicide prevention education experiences into their curriculum for second year trainees. Our proposed training timelines for both conditions and learner groups are designed to mirror real-world circumstances where providers are not likely to encounter a patient at risk for suicide immediately following video-based expert teaching in suicide prevention. Therefore, we planned SP practice encounters for the experimental group within four months of completing video-based modules so that both the experimental and control groups could experience the “test case” at around 6-month follow-up. However, many of the residency rotations are not able to support time for the resident-SP encounters, which has posed challenges for scheduling SP sessions for the experimental participants. Four rotations relevant to suicide prevention (i.e., adolescent outpatient, ambulatory pediatrics, developmental behavioral pediatrics, practice-based elective rotations) offered times to complete SP sessions. So far, timing of these rotations for some residents in the experimental group has been less consistent with the study timeline. Residents in the control group pose far fewer logistical challenges.

Participants in both conditions are aware that the simulated patient interaction is part of the suicide prevention training program. Therefore, bias is not likely to impact condition comparisons because it is the same for all participants. We acknowledge, however, that trainees are likely to be more alert to suicide risk and management than in usual practice.

Rigorous observational coding of participant suicide prevention skills is underway. However, we have already realized that we similarly need to measure actor-educator fidelity to the SP character and feedback procedures. Although the 14 SPs are rigorously trained and tested for adherence to character and procedural standards prior to working in the study, there is likely to be variability in their interactions. Additionally, there may be drift in adherence to standards over the course of the study. A fidelity measure (noted in the measures section above) has been developed and added to the coding efforts which will enable us to analyze for the effect of actor fidelity on participants’ demonstrated skills.

Finally, during our first group of subjects, we learned that several participants from the nurse practitioner program attended an unrelated seminar on suicide prevention, which occurred while they were completing activities for our training program. This prompted us to add an item to the final training survey that asked participants how many hours of additional (i.e., not including our training) concurrent suicide prevention training they received since beginning the training. Doing so allows us to account for possible skill improvement due to other parallel training.

## Abbreviations

CTL-PC: Commitment to Living for Primary Care
PCP: primary care provider
SP: standardized patient
